# 6-Bromo-2-methyl­sulfanyl-1,3-benzo­thia­zole

**DOI:** 10.1107/S160053681105015X

**Published:** 2011-11-25

**Authors:** Michał A. Dobrowolski, Marta Struga, Daniel Szulczyk

**Affiliations:** a University of Warsaw, Faculty of Chemistry, Pasteura 1, 02-093 Warsaw, Poland; b Medical University of Warsaw, Faculty of Medicine, Oczki 3, 02-007 Warsaw, Poland

## Abstract

The title mol­ecule, C_8_H_6_BrNS_2_, is almost planar with a dihedral angle of 0.9 (1)° between the benzene and thia­zole rings. The values of the geometry-based index of aromaticity (HOMA) and the nucleus-independent chemical shift (NICS) for the two cyclic fragments of the title mol­ecule are 0.95 and −9.61, respectively, for the benzene ring, and 0.69 and −7.71, respectively, for the thia­zole ring. They show that the benzene ring exhibits substanti­ally higher cyclic π-electron delocalization than the thia­zole ring. Comparison with other similar benzothia­zole fragments reveals a similar trend.

## Related literature

For a description of the Cambridge Structural Database, see: Allen (2002 ▶). For related structures, see: Chen *et al.* (2003 ▶, 2010 ▶); Li *et al.* (2009 ▶); Liu *et al.* (2003 ▶); Loghmani-Khouzani *et al.* (2009 ▶); Matthews *et al.* (1996 ▶); Saravanan *et al.* (2007 ▶); Zhao *et al.* (2009 ▶); Zou *et al.* (2003 ▶). For the aromaticity of benzothia­zoles, see: Karolak-Wojciechowska *et al.* (2007 ▶). For the Gaussian program, see: Frisch *et al.* (2009 ▶). For the HOMA index, see: Kruszewski & Krygowski (1972 ▶); Krygowski & Cyrański (2001 ▶) and for the NICS index, see: Schleyer *et al.* (1996 ▶).
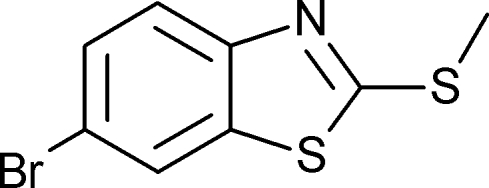


## Experimental

### 

#### Crystal data

C_8_H_6_BrNS_2_
*M* *_r_* = 260.18
Monoclinic, 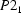

*a* = 9.7843 (4) Å
*b* = 3.9514 (2) Å
*c* = 11.6076 (5) Å
β = 96.353 (4)°
*V* = 446.01 (3) Å^3^
*Z* = 2
Mo *K*α radiation
μ = 5.01 mm^−1^
*T* = 100 K
0.4 × 0.15 × 0.1 mm

#### Data collection

Oxford Diffraction Xcalibur S diffractometer
Absorption correction: multi-scan (*CrysAlis RED*; Oxford Diffraction, 2009 ▶) *T*_min_ = 0.422, *T*_max_ = 0.606
3437 measured reflections
1227 independent reflections
1125 reflections with *I* > 2σ(*I*)
*R*_int_ = 0.024

#### Refinement

*R*[*F* ^2^ > 2σ(*F* ^2^)] = 0.017
*wR*(*F* ^2^) = 0.033
*S* = 0.99
1227 reflections
110 parameters
1 restraint
H-atom parameters constrained
Δρ_max_ = 0.36 e Å^−3^
Δρ_min_ = −0.22 e Å^−3^
Absolute structure: Flack (1983 ▶), 315 Friedel pairs
Flack parameter: 0.005 (9)

### 

Data collection: *CrysAlis CCD* (Oxford Diffraction, 2009 ▶); cell refinement: *CrysAlis RED* (Oxford Diffraction, 2009 ▶); data reduction: *CrysAlis RED*; program(s) used to solve structure: *SHELXS97* (Sheldrick, 2008 ▶); program(s) used to refine structure: *SHELXL97* (Sheldrick, 2008 ▶); molecular graphics: *Mercury* (Macrae *et al.*, 2006 ▶); software used to prepare material for publication: *publCIF* (Westrip, 2010 ▶).

## Supplementary Material

Crystal structure: contains datablock(s) I, global. DOI: 10.1107/S160053681105015X/gk2424sup1.cif

Structure factors: contains datablock(s) I. DOI: 10.1107/S160053681105015X/gk2424Isup2.hkl

Supplementary material file. DOI: 10.1107/S160053681105015X/gk2424Isup3.cml

Additional supplementary materials:  crystallographic information; 3D view; checkCIF report

## Figures and Tables

**Table 1:** HOMA indices for compounds containing benzothia­zole moieties. BT = benzothia­zole; MePyr = methyl­pyridine.

refcode	*R* =	HOMA (total)	HOMA (thia­zole)	HOMA (benzene)
This work	H	0.82	0.69	0.95
DIDBAU*^*a*^*	-C(Ph)=N—NH—C(O)—NH2	0.85	0.73	0.99
HUFSIL*^*b*^*	-CH2—O—CH2—CH2—S—BT	0.83	0.69	0.98
HUYYIJ*^*c*^*	-CH2—CH2—CH2—S—BT	0.84	0.70	0.98
MACMOT*^*d*^*	-CH2—S—BT	0.85	0.72	0.97
	-CH2—S—BT	0.85	0.71	0.98
MACMOT01*^*e*^*	-CH2—S—BT	0.85	0.70	0.98
	-CH2—S—BT	0.85	0.71	0.97
MOKJIG*^*f*^*	-C(O)—C(COOCH3)=N—O—CH3	0.83	0.70	0.96
PUFGED*^*g*^*	-C(O)—Ph	0.84	0.70	0.96
QOTQAS*^*h*^*	-C(O)—NH-2-MePyr	0.85	0.71	0.98
ZUQQEH^*i*^	-CH2—O—CH2—CH2—O– CH2—CH2—S—BT	0.83	0.67	0.98
Mean		0.84	0.70	0.97
E.s.d.		0.01	0.01	0.01

Notes: (*a*) Saravanan *et al.* (2007 ▶); (*b*) Chen *et al.* (2010 ▶); (*c*) Chen *et al.* (2003 ▶); (*d*) Liu *et al.* (2003 ▶); (*e*) Zou *et al.* (2003 ▶); (*f*) Li *et al.* (2009 ▶); (*g*) Loghmani-Khouzani *et al.* (2009 ▶); (*h*) Matthews *et al.* (1996 ▶); (*i*) Zhao *et al.* (2009 ▶).

**Fig. 1.**
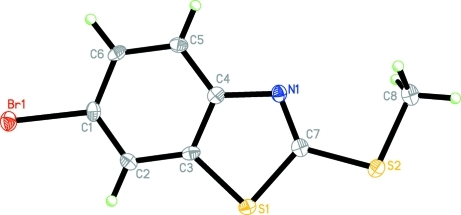
The molecular structure of the title compound showing displacement ellipsoids at the 50% probability level.

**Fig. 2.**
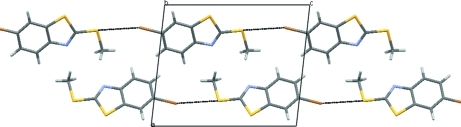
Short contacts between bromine and sulfur atoms; view along the b axis. Intermolecular Br···S are shown with dashed lines.

## supplementary crystallographic information

### Comment

Our report concerns 6-bromo-2-(methylthio)benzo[*d*]thiazole (Fig.1). Its structure is essentially planar with dihedral angle between conjugated thiazole and benzene rings equal to 1.0 (1)°. Random deviation from 5-membered ring plane is 0.0019 Å. This is very similar to previously investigated systems containing benzothiazole moiety (Karolak-Wojciechowska *et al.*, 2007).

There are four C—S bonds present in the molecule, which are formally single. However, except for C8—S2 [1.805 (3) Å ; reference bond length for single C—S bond used for HOMA is 1.807 Å, Krygowski & Cyrański (2001)], three other bonds are shorter [C7—S2 1.744 (3) Å, C7—S1 1.760 (3) Å, C3—S1 1.730 (3) Å, Fig 1]. This results from π···π conjugation and leads to higher cyclic delocalization in the thiazole ring. There are no hydrogen bonds present, but a short contact of 3.6133 (9) Å between bromine and sulfur atoms is observed (Fig. 2).

It has been shown for benzothiazoles that global aromaticity is always higher than for the single five-membered ring (Karolak-Wojciechowska *et al.*, 2007). This is the consequence of the thiazole ring conjugation with fully aromatic benzene ring.

To estimate cyclic π-electron delocalization in our system we used geometry based Harmonic Oscillator Model of Aromaticity. HOMA can be calculated for both the whole molecule or individual moiety. For the title benzothiazole moiety HOMA equals 0.82, whereas for thiazole ring 0.69 and 0.95 for benzene ring (Table 1). The value of HOMA for benzene ring is higher than for thiazole, what is additionally corroborated by the magnetic indices of aromaticity. For thiazole ring NICS(0) = -7.71, NICS(1)zz = -15.89. For benzene ring NICS(0) = -9.61, NICS(1)_zz_ = -25.44. For comparison we calculated HOMA indices for other 9 compounds derived from the Cambridge Structural Database (Allen, 2002) containing benzothiazole fragment. All of them showed similar trend. Selected compounds had to fulfill the following criteria: (i) *R* factor below 5%, (ii) only the carbon atom of the methylthio group bears a substituent and this substituent binds through the carbon atom, (iii) benzothiazole moiety is not embedded in heterocyclic ring. The values of indices are gathered in Table 1. HOMA values indicate large π-electron delocalization in benzene ring in all compounds. Thiazole ring shows lower aromaticity than the global HOMA in all cases. Noteworthy, the variation of indices for both fragments is very small, but it is fair to note that the structure modifications are not important as only the substituents joined to the carbon atom of methylthio group change.

### Experimental

To a 5.01 g (0.025 mol) of 2-(methylthio)benzo[*d*]thiazole suspended in 50 ml of methanol the 1.5 ml of pure Br2 was added dropwise in portions (5 drops every 20 min). Since the beginning of the reaction the solution was extensively stirred for 8 h, and then obtained precipitate (4.67 g, 93%) was separated from reaction mixture and washed with ice cold methanol (3 portions of 50 ml). White solid residue was crystallized from absolute ethanol. Melting point 98-101°C. Single crystals were obtained immediately after slow evaporation of ethanol.

The calculations were carried out using Gaussian09 program (Frisch *et al.*, 2009), starting from the X-ray geometry. Effective core potential for the bromine atom at the B3LYP/LANL2DZ theoretical level was used. NICS values were computed at GIAO/B3LYP/6–311+G**; the NICS(1) points are 1 Å above ring centres, perpendicular to the averaged planes of the rings. HOMA indices were calculated using personal program by one of the authors (M. A. D.).

### Refinement

H atoms were placed in calculated positions with C—H = 0.95–0.98 Å, and refined in riding mode with *Uiso*(H) = 1.2–1.5 *Ueq*(C).

### Crystal data

**Table:**

C_8_H_6_BrNS_2_	*F*(000) = 256
*M**_r_* = 260.18	*D*_x_ = 1.937 Mg m^−^^3^
Monoclinic, *P*2_1_	Melting point = 371–374 K
Hall symbol: P 2yb	Mo *K*α radiation, λ = 0.71073 Å
*a* = 9.7843 (4) Å	Cell parameters from 2764 reflections
*b* = 3.9514 (2) Å	θ = 2.9–28.5°
*c* = 11.6076 (5) Å	µ = 5.01 mm^−^^1^
β = 96.353 (4)°	*T* = 100 K
*V* = 446.01 (3) Å^3^	Needle, colourless
*Z* = 2	0.4 × 0.15 × 0.1 mm

### Data collection

**Table:**

Oxford Diffraction Xcalibur S diffractometer	1227 independent reflections
Radiation source: fine-focus sealed tube	1125 reflections with *I* > 2σ(*I*)
graphite	*R*_int_ = 0.024
Detector resolution: 8.6479 pixels mm^-1^	θ_max_ = 25.0°, θ_min_ = 2.9°
phi and ω scans	*h* = −11→11
Absorption correction: multi-scan (*CrysAlis RED*; Oxford Diffraction, 2009)	*k* = −4→3
*T*_min_ = 0.422, *T*_max_ = 0.606	*l* = −13→13
3437 measured reflections	

### Refinement

**Table:**

Refinement on *F*^2^	Secondary atom site location: difference Fourier map
Least-squares matrix: full	Hydrogen site location: inferred from neighbouring sites
*R*[*F*^2^ > 2σ(*F*^2^)] = 0.017	H-atom parameters constrained
*wR*(*F*^2^) = 0.033	*w* = 1/[σ^2^(*F*_o_^2^) + (0.0143*P*)^2^] where *P* = (*F*_o_^2^ + 2*F*_c_^2^)/3
*S* = 0.99	(Δ/σ)_max_ = 0.002
1227 reflections	Δρ_max_ = 0.36 e Å^−^^3^
110 parameters	Δρ_min_ = −0.22 e Å^−^^3^
1 restraint	Absolute structure: Flack (1983), 315 Friedel pairs
Primary atom site location: structure-invariant direct methods	Flack parameter: 0.005 (9)

### Special details

**Table:**

Geometry. All e.s.d.'s (except the e.s.d. in the dihedral angle between two l.s. planes) are estimated using the full covariance matrix. The cell e.s.d.'s are taken into account individually in the estimation of e.s.d.'s in distances, angles and torsion angles; correlations between e.s.d.'s in cell parameters are only used when they are defined by crystal symmetry. An approximate (isotropic) treatment of cell e.s.d.'s is used for estimating e.s.d.'s involving l.s. planes.
Refinement. Refinement of *F*^2^ against ALL reflections. The weighted *R*-factor *wR* and goodness of fit *S* are based on *F*^2^, conventional *R*-factors *R* are based on *F*, with *F* set to zero for negative *F*^2^. The threshold expression of *F*^2^ > σ(*F*^2^) is used only for calculating *R*-factors(gt) *etc*. and is not relevant to the choice of reflections for refinement. *R*-factors based on *F*^2^ are statistically about twice as large as those based on *F*, and *R*- factors based on ALL data will be even larger.

### Fractional atomic coordinates and isotropic or equivalent isotropic displacement parameters (Å^2^)

**Table:**

	*x*	*y*	*z*	*U*_iso_*/*U*_eq_	
C1	0.7671 (3)	0.6254 (7)	0.9919 (3)	0.0141 (7)	
C2	0.8559 (3)	0.5366 (12)	0.9147 (2)	0.0123 (6)	
C3	0.8181 (3)	0.6157 (7)	0.7984 (3)	0.0121 (7)	
C4	0.6939 (3)	0.7815 (8)	0.7626 (3)	0.0121 (7)	
C5	0.6060 (3)	0.8712 (7)	0.8439 (3)	0.0137 (7)	
C6	0.6430 (3)	0.7938 (8)	0.9590 (3)	0.0135 (7)	
C7	0.7722 (3)	0.7402 (8)	0.5934 (3)	0.0127 (7)	
C8	0.6257 (3)	0.9551 (10)	0.3940 (3)	0.0192 (8)	
N1	0.6702 (3)	0.8486 (6)	0.6440 (2)	0.0131 (6)	
S1	0.90775 (7)	0.5420 (3)	0.68048 (6)	0.01509 (19)	
S2	0.79094 (8)	0.7776 (2)	0.44632 (7)	0.01761 (19)	
Br1	0.81246 (3)	0.50973 (9)	1.15124 (2)	0.01701 (9)	
H2	0.9403	0.4254	0.9390	0.015*	
H5	0.5218	0.9841	0.8205	0.016*	
H6	0.5845	0.8546	1.0158	0.016*	
H8A	0.6084	1.1556	0.4399	0.029*	
H8B	0.5537	0.7867	0.4011	0.029*	
H8C	0.6254	1.0196	0.3124	0.029*	

### Atomic displacement parameters (Å^2^)

**Table:**

	*U*^11^	*U*^22^	*U*^33^	*U*^12^	*U*^13^	*U*^23^
C1	0.0180 (18)	0.0130 (17)	0.0109 (16)	−0.0034 (13)	−0.0003 (14)	0.0000 (13)
C2	0.0133 (13)	0.0076 (16)	0.0157 (14)	−0.0013 (16)	0.0000 (10)	0.0028 (16)
C3	0.0103 (16)	0.0095 (15)	0.0169 (16)	−0.0012 (12)	0.0029 (13)	−0.0044 (12)
C4	0.0120 (17)	0.0099 (16)	0.0144 (17)	−0.0050 (14)	0.0012 (13)	−0.0032 (14)
C5	0.0122 (17)	0.0110 (15)	0.0178 (18)	0.0005 (12)	0.0013 (13)	−0.0010 (13)
C6	0.0134 (17)	0.0123 (16)	0.0157 (18)	−0.0017 (14)	0.0051 (13)	−0.0050 (14)
C7	0.0137 (17)	0.0090 (15)	0.0150 (17)	−0.0048 (14)	−0.0001 (14)	−0.0006 (14)
C8	0.0213 (17)	0.019 (2)	0.0171 (16)	−0.0014 (17)	−0.0006 (13)	0.0025 (17)
N1	0.0132 (15)	0.0136 (13)	0.0122 (14)	−0.0018 (11)	0.0011 (11)	0.0012 (11)
S1	0.0140 (4)	0.0166 (5)	0.0149 (4)	0.0027 (5)	0.0027 (3)	−0.0012 (5)
S2	0.0190 (5)	0.0197 (4)	0.0149 (4)	0.0020 (4)	0.0049 (3)	0.0010 (4)
Br1	0.02064 (16)	0.01663 (14)	0.01365 (15)	0.0003 (2)	0.00146 (11)	0.0021 (2)

### Geometric parameters (Å, °)

**Table:**

S1—C3	1.730 (3)	C6—H6	0.9500
S1—C7	1.760 (3)	C3—C4	1.402 (4)
N1—C7	1.286 (4)	C4—C5	1.391 (4)
N1—C4	1.396 (4)	C5—H5	0.9500
C2—C1	1.362 (4)	C7—S2	1.744 (3)
C2—C3	1.395 (4)	S2—C8	1.805 (3)
C2—H2	0.9500	C8—H8A	0.9800
C1—C6	1.400 (4)	C8—H8B	0.9800
C1—Br1	1.909 (3)	C8—H8C	0.9800
C6—C5	1.379 (4)		
			
C3—S1—C7	87.90 (14)	C5—C4—C3	119.9 (3)
C7—N1—C4	109.5 (3)	N1—C4—C3	115.2 (3)
C1—C2—C3	117.3 (3)	C6—C5—C4	119.0 (3)
C1—C2—H2	121.3	C6—C5—H5	120.5
C3—C2—H2	121.3	C4—C5—H5	120.5
C2—C1—C6	122.7 (3)	N1—C7—S2	126.2 (2)
C2—C1—Br1	118.6 (2)	N1—C7—S1	117.4 (2)
C6—C1—Br1	118.7 (2)	S2—C7—S1	116.46 (18)
C5—C6—C1	119.8 (3)	C7—S2—C8	100.09 (15)
C5—C6—H6	120.1	S2—C8—H8A	109.5
C1—C6—H6	120.1	S2—C8—H8B	109.5
C2—C3—C4	121.3 (3)	H8A—C8—H8B	109.5
C2—C3—S1	128.6 (2)	S2—C8—H8C	109.5
C4—C3—S1	110.0 (2)	H8A—C8—H8C	109.5
C5—C4—N1	124.9 (3)	H8B—C8—H8C	109.5
			
C3—C2—C1—C6	−0.9 (5)	C2—C3—C4—N1	179.2 (3)
C3—C2—C1—Br1	178.1 (3)	S1—C3—C4—N1	0.1 (3)
C2—C1—C6—C5	1.0 (5)	C1—C6—C5—C4	−0.4 (4)
Br1—C1—C6—C5	−178.1 (2)	N1—C4—C5—C6	−179.0 (3)
C1—C2—C3—C4	0.3 (5)	C3—C4—C5—C6	−0.1 (4)
C1—C2—C3—S1	179.3 (3)	C4—N1—C7—S2	−178.1 (2)
C7—S1—C3—C2	−178.8 (4)	C4—N1—C7—S1	0.6 (3)
C7—S1—C3—C4	0.2 (2)	C3—S1—C7—N1	−0.5 (3)
C7—N1—C4—C5	178.5 (3)	C3—S1—C7—S2	178.36 (19)
C7—N1—C4—C3	−0.4 (4)	N1—C7—S2—C8	−6.0 (3)
C2—C3—C4—C5	0.2 (5)	S1—C7—S2—C8	175.2 (2)
S1—C3—C4—C5	−178.9 (2)		

### Table 1 HOMA indices for compounds containing benzothiazole moieties.

BT = benzothiazole; MePyr = methylpyridine.

**Table:**

refcode	*R* =	HOMA (total)	HOMA (thiazole)	HOMA (benzene)
This work	H	0.82	0.69	0.95
DIDBAU*^a^*	-C(Ph)=N-NH-C(O)-NH2	0.85	0.73	0.99
HUFSIL*^b^*	-CH2-O-CH2-CH2-S-BT	0.83	0.69	0.98
HUYYIJ*^c^*	-CH2-CH2-CH2-S-BT	0.84	0.70	0.98
MACMOT*^d^*	-CH2-S-BT	0.85	0.72	0.97
	-CH2-S-BT	0.85	0.71	0.98
MACMOT01*^e^*	-CH2-S-BT	0.85	0.70	0.98
	-CH2-S-BT	0.85	0.71	0.97
MOKJIG*^f^*	-C(O)-C(COOCH3)=N-O-CH3	0.83	0.70	0.96
PUFGED*^g^*	-C(O)-Ph	0.84	0.70	0.96
QOTQAS*^h^*	-C(O)-NH-2-MePyr	0.85	0.71	0.98
ZUQQEH*^i^*	-CH2-O-CH2-CH2-O- CH2-CH2-S-BT	0.83	0.67	0.98
Mean		0.84	0.70	0.97
E.s.d.		0.01	0.01	0.01

Notes: (*a*) Saravanan *et al.* (2007); (*b*) Chen *et al.* (2010); (*c*) Chen *et al.* (2003); (*d*) Liu *et al.* (2003); (*e*) Zou *et al.* (2003); (*f*) Li *et al.* (2009); (*g*) Loghmani-Khouzani *et al.* (2009); (*h*) Matthews *et al.* (1996); (*i*) Zhao *et al.* (2009).
